# High-resolution cone-beam CT (DynaCT Micro) enhances preoperative and intraoperative visualization of intracranial perforator arteries

**DOI:** 10.1007/s00234-026-04052-2

**Published:** 2026-06-03

**Authors:** Mikaeel A. Habib, Jaeha Kim, William J. Lee, Luis O. Tierradentro-Garcia, Om H. Gandhi, Robert Romano, Suehyb G. Alkhatib, Daniel Gewolb, Colbey Freeman, Sarah A. Hamimi, Mackenzie Castellanos, Sami Almasri, Linda J. Bagley, Omar A. Choudhri

**Affiliations:** 1 Department of Neurosurgery, University of Pennsylvania, Philadelphia, PA USA; 2 Radiology, University of Pennsylvania, Philadelphia, PA USA; 3 Radiology Associates of Richmond, Richmond, VA USA; 4 Department of Radiology, University of Miami, Coral Gables, FL USA

**Keywords:** High-resolution cone-beam CT, Intracranial perforator arteries, Intraoperative imaging, CT angiography, Cerebrovascular intervention

## Abstract

**Background and purpose:**

Preservation of perforator arteries during cerebrovascular interventions remains challenging due to their submillimeter caliber and variable anatomy. High-resolution cone-beam computed tomography (HR-CBCT) offers improved resolution compared with conventional CT angiography (CTA), yet its utility for perforator assessment has not been systematically evaluated. This study assessed HR-CBCT for visualization of intracranial perforators in both preoperative and intraoperative contexts.

**Methods:**

Imaging from 20 patients who underwent aneurysm clipping, arteriovenous malformation resection, or dural arteriovenous fistula treatment between July 2022 and November 2024 was reviewed. Three imaging modalities were evaluated: preoperative CTA, preoperative HR-CBCT, and intraoperative HR-CBCT. Five perforator arteries—the recurrent artery of Heubner, anterior choroidal artery, lateral lenticulostriate arteries, basilar artery perforators, and posterior cerebral artery perforators—were independently scored by four neuroradiologists using a three-point visibility scale (3 = visible, 2 = not visible due to limited field of view, 1 = not visible due to resolution). Reader confidence was recorded, and statistical analysis was performed using R.

**Results:**

Perforator visualization was significantly higher with both preoperative and intraoperative HR-CBCT compared with CTA. On preoperative imaging, confidence was significantly higher for anterior choroidal and lateral lenticulostriate arteries (*p* < 0.001), but not for the recurrent artery of Heubner (*p* = 0.102). Intraoperative scans showed no significant reduction in visibility. Effective dose did not differ between CTA and HR-CBCT (*p* = 0.700).

**Conclusions:**

Preoperative and intraoperative HR-CBCT provide superior perforator visualization compared with CTA, with intraoperative imaging closely matching preoperative performance.

**Supplementary Information:**

The online version contains supplementary material available at https://doi.org/10.1007/s00234-026-04052-2.

## Introduction

Intraoperative imaging has transformed cerebrovascular surgery by enabling real-time visualization of cerebrovascular anatomy, which is critical for both surgical precision and intraoperative decision-making [1]. Its role is particularly important during procedures such as aneurysm clipping, arteriovenous malformation (AVM) resection, and dural arteriovenous fistula (dAVF) treatment, where morbidity rates from the procedure can range from 2.2% to 13.7% [2]. Most of the post-operative complications stem from ischemic complications, where intervention causes unintended occlusion of downstream vessels [2]. This underscores the imperative to develop a robust imaging modality that can detect these perforators pre-operatively and potentially demonstrate occlusions intraoperatively.

Intraoperative angiography has been shown to improve clip placement accuracy and reduce rates of residual aneurysm or large-vessel occlusion [3]. Nevertheless, prior studies have also documented its limitations in failing to detect residual aneurysms or branch vessel compromise and its inability to prevent ischemic complications resulting from inadvertent injury to small perforator vessels [3]. 

Perforator arteries are particularly important in this context because they supply deep and highly eloquent brain structures [4]. Among the anterior perforators, some of the most clinically significant include the recurrent artery of Heubner (RAH), which supplies portions of the caudate, putamen, and anterior limb of the internal capsule; the lateral lenticulostriate arteries (LSAs), which supply the basal ganglia and large portions of the internal capsule; and the anterior choroidal artery (AChA), which supplies the posterior limb of the internal capsule, optic tract, and medial globus pallidus [4]. The damage or occlusion of these small vessels during neurosurgical procedures such as microsurgical clipping, embolization, or stenting can result in perforator infarctions, and even a single infarct in their distribution may cause profound and permanent deficits, including hemiplegia, sensory loss, or visual field disturbances [4–7]. 

Preserving these perforators is therefore essential during cerebrovascular interventions, yet their submillimeter caliber and variable anatomy make them notoriously difficult to visualize [4]. Currently, operative visualization of perforators under the microscope and neurophysiologic monitoring are the two tools generally used to confirm perforator preservation albeit in an imperfect fashion [8]. Despite these tools, 77% of perforator infarcts after microsurgical clipping happen without direct injury to perforator vessels, with neurophysiologic monitoring changes seen in only 5–7% of these cases [9]. 

Among available intraoperative imaging modalities, high-resolution cone-beam computed tomography (HR-CBCT; DynaCT Micro, Siemens) has emerged as a technique capable of visualizing submillimeter intracranial microvasculature with exceptional detail [5, 10]. Cone-beam computed tomography (CBCT) utilizes a mobile C-arm system that acquires several X-ray projections around a volume of interest using a gantry rotation, utilizing post-processing algorithms to reconstruct a 3D image [11, 12]. HR-CBCT utilizes improved image processing algorithms, post-processing, and contrast to improve spatial resolution to 0.15 mm, making it a more ideal imaging method to visualize small vasculature [13]. Beyond its ability to detect perforators, this modality aids in the precise localization of vascular pathologies and delineation of critical neurovascular structures, which is especially important during microsurgical clipping.

Intraoperative digital subtraction angiography (DSA) remains the current gold standard for confirming aneurysm occlusion and large-vessel patency and is also used preoperatively to identify perforators [1]. However, prior studies have shown that HR-CBCT provides superior perforator visualization compared with both three-dimensional rotational angiography (3D-RA) and conventional two-dimensional DSA (2D-DSA) [5, 10]. This capability raises the possibility of intraoperative HR-CBCT being used not only for anatomic depiction but also for real-time assessment of vessel patency after clipping, coiling, or stenting, potentially reducing the risk of perforator infarctions and improving surgical outcomes.

Building upon this foundation, the present study represents a single-center, retrospective feasibility analysis comparing preoperative HR-CBCT, intraoperative HR-CBCT, and conventional preoperative CT angiography (CTA) for visualization of cerebral perforating arteries. Given the exploratory nature of this investigation and the relatively small cohort (20 total patients; 10 preoperative HR-CBCT studies, 13 CTA studies, and 20 intraoperative HR-CBCT acquisitions), the study was designed to evaluate imaging performance rather than clinical outcomes. We surveyed neuroradiologists with varying levels of experience and focused on key anterior circulation perforators as well as selected posterior circulation perforators when applicable.

The primary endpoint was perforator visibility on each modality, assessed independently per reader. Secondary endpoints included reader-reported diagnostic confidence in perforator identification and comparison of radiation dose metrics across modalities. No clinical outcome data (perforator infarction, procedural complications, or functional outcomes) were analyzed. The objective was to evaluate feasibility, inter-reader reliability, and the potential procedural utility of intraoperative HR-CBCT as a real-time adjunct during cerebrovascular interventions.

## Materials and methods

### Subjects

This retrospective study included 20 patients who underwent aneurysm clipping (*n* = 14), arteriovenous malformation resection (*n* = 4), or dural arteriovenous fistula treatment (*n* = 2) at our institution. The mean age of participants at first imaging was 55.3 years (26.4–73.7 years, standard deviation 14.8). This retrospective, observational study was approved by the Institutional Review Board of our institution, and a waiver of written informed consent was granted. This study adhered to the Strengthening the Reporting of Observational Studies in Epidemiology (STROBE) guidelines for observational studies (Table S1) [14]. 

### Imaging acquisition

High-resolution cone-beam computed tomography (HR-CBCT; DynaCT Micro) was obtained preoperatively and intraoperatively using a 40 × 30-cm Siemens ARTIS icono biplane flat-panel detector system. Acquisition parameters were standardized across all scans: 108.2 kV x-ray voltage, 14-second rotation, 200° total angulation, 0.4° angular increment, and 496 projections for volumetric reconstruction with a 25-cm zoom format. Contrast (Isovue-300) was diluted to 70% (selected to limit excessive intraluminal opacification, which can otherwise obscure adjacent metallic hardware such as surgical clips) and injected at 2 mL/s for a total of 34 mL using a Nemoto Duo Press Elite injector (Nemoto Kyorindo Co., Tokyo, Japan), with a fixed 3-second x-ray delay applied uniformly across cases and no target-specific modification of injection timing. For all HR-CBCT studies, contrast was administered through a diagnostic catheter positioned in the internal carotid or vertebral artery depending on target anatomy. Preoperative HR-CBCT studies were performed under monitored anesthesia care. The Siemens ARTIS ICONO biplane angiography system was used in the hybrid operating room setting for intraoperative HR-CBCT imaging. The timing of intraoperative HR-CBCT acquisition varied by case. In some patients the dura had been closed and the bone flap replaced before imaging, while in others, particularly when residual aneurysm or arteriovenous malformation could not be excluded with confidence, acquisition was performed prior to bone flap replacement. The surgical field was irrigated with saline before each acquisition to reduce air artifact at the cortical surface. No qualitative difference in image quality was observed between scans obtained with and without the bone flap in place.

Preoperative CTA was performed on a multidetector CT scanner (Revolution CT, GE Medical Systems, Milwaukee, WI) using institutional cerebrovascular protocol. Images were acquired at 120 kVp with automated tube-current modulation and reconstructed at 0.625-mm slice thickness using a standard convolution kernel and a 236-mm field of view (512 × 512 matrix), yielding an in-plane resolution of approximately 0.46 mm.

All scans underwent a quality review. Exclusion criteria included motion artifact, inadequate contrast opacification, incomplete field of view, and—in the case of HR-CBCT—incomplete rotation or streak artifact obscuring the region of interest. No scans were excluded on quality grounds. A total of 10 preoperative HR-CBCT, 13 CTA, and 20 intraoperative HR-CBCT scans met inclusion criteria for analysis. Some patients had multiple scans across different imaging modalities.

Images were reviewed on the Sectra IDS7 workstation (Sectra AB, Linköping, Sweden; version 25.2) using default MIP and MPR settings, with slab thicknesses of 1.5–2 mm (most cases 2 mm) and interspace gaps of 0.8–1.0 mm. Images were evaluated in all three planes: axial, sagittal, and coronal. Pixel spacing varied among images but ranged 0.035–0.11 mm.

### Imaging presentation and blinding

Images were distributed to readers in a survey-based format for independent review. No clinical history or patient demographic information was provided. Cases were presented in sequential chronological order (oldest to most recent) and were not randomized. Imaging modality was not explicitly labeled; however, due to inherent differences in image characteristics between CTA and HR-CBCT, complete blinding to modality may not have been possible.

### Neuroradiologist scoring

Perforator visualization was assessed by four CAQ-certified neuroradiologists (Readers A–D) stratified into three levels of experience (PGY-6, PGY-8, PGY-34). Each reader independently reviewed preoperative HR-CBCT, intraoperative HR-CBCT, and preoperative CTA scans and evaluated five perforator vessels: recurrent artery of Heubner (RAH), anterior choroidal artery (AChA), lateral lenticulostriate arteries (LSAs), basilar artery perforators (BAPs), and posterior cerebral artery perforators (PCAPs). Vessels were categorized as visible, not visible due to restricted field of view, or not visible due to resolution limitations. Readers also denoted scans that were not applicable or should otherwise be excluded from analysis. Reader confidence for each identification was scored on a four-point scale (1 = not confident, 2 = slightly confident, 3 = confident, 4 = very confident). RAH, AChA, LSAs were designated as primary outcome vessels for comparative analysis, whereas BAPs and PCAPs were considered exploratory and excluded from the final comparative analysis due to a limited number of evaluable scans. Percentages are represented as a proportion of per-reader-evaluations rather than per-scan frequencies. Readers based their assessments on image features relevant to named vessel identification and vessel delination, including perceived spatial resolution, signal-to-noise ratio (SNR), contrast-to-noise ratio (CNR), and vessel-to-background contrast.

### Inter-reader reliability

Inter-reader agreement for perforating artery visualization was assessed across four neuroradiologists (two fellowship-trained attendings and two senior fellows) using Fleiss’ κ and Gwet’s AC1 (Table S4). Vessel visibility was binarized as visualized versus not visualized for each scan. Because near-unanimous agreement on a single category can produce paradoxically low κ values (the “kappa paradox”), AC1 was used as the primary agreement statistic, as it is robust to prevalence-driven bias [15–17]. Reader confidence (rated 1–4) was evaluated using the two-way random, single measures, absolute agreement, intraclass correlation coefficient [ICC [1, 2] ], and Gwet’s AC2 with quadratic weights.

### Calculating effective dosages

Radiation exposure data for CTA and HR-CBCT was obtained from post-operative dose logs. Radiation data from CTA was represented as dose-length product (DLP, mGy-cm) and dose-area product (DAP, mSv/mGy-cm^2^). In order to make fair comparisons between the two imaging modalities, effective dosages of radiation, in mSv, were calculated using ICRP-derived conversion factors (k = 0.0025 mSv/mGy-cm, 0.0056 mSv/mGy-cm^2^) [18]. 

### Statistical analysis

All statistical analyses were performed using R (version 4.4.2). Patient demographics and clinical characteristics were compared across imaging modality groups using a two-sided Fisher exact test for categoric variables or the Wilcoxon rank-sum test. Perforator vessel visualization was compared across imaging modalities utilizing Chi-square test of independence with post-hoc Bonferroni correction. Comparisons of reader confidence of vessel visualization was performed using the Kruskal-Wallis test with post-hoc Dunn’s test for pairwise comparisons. Comparisons of radiation doses were conducted using the Mann-Whitney U test.

## Results

**Fig. 1:**
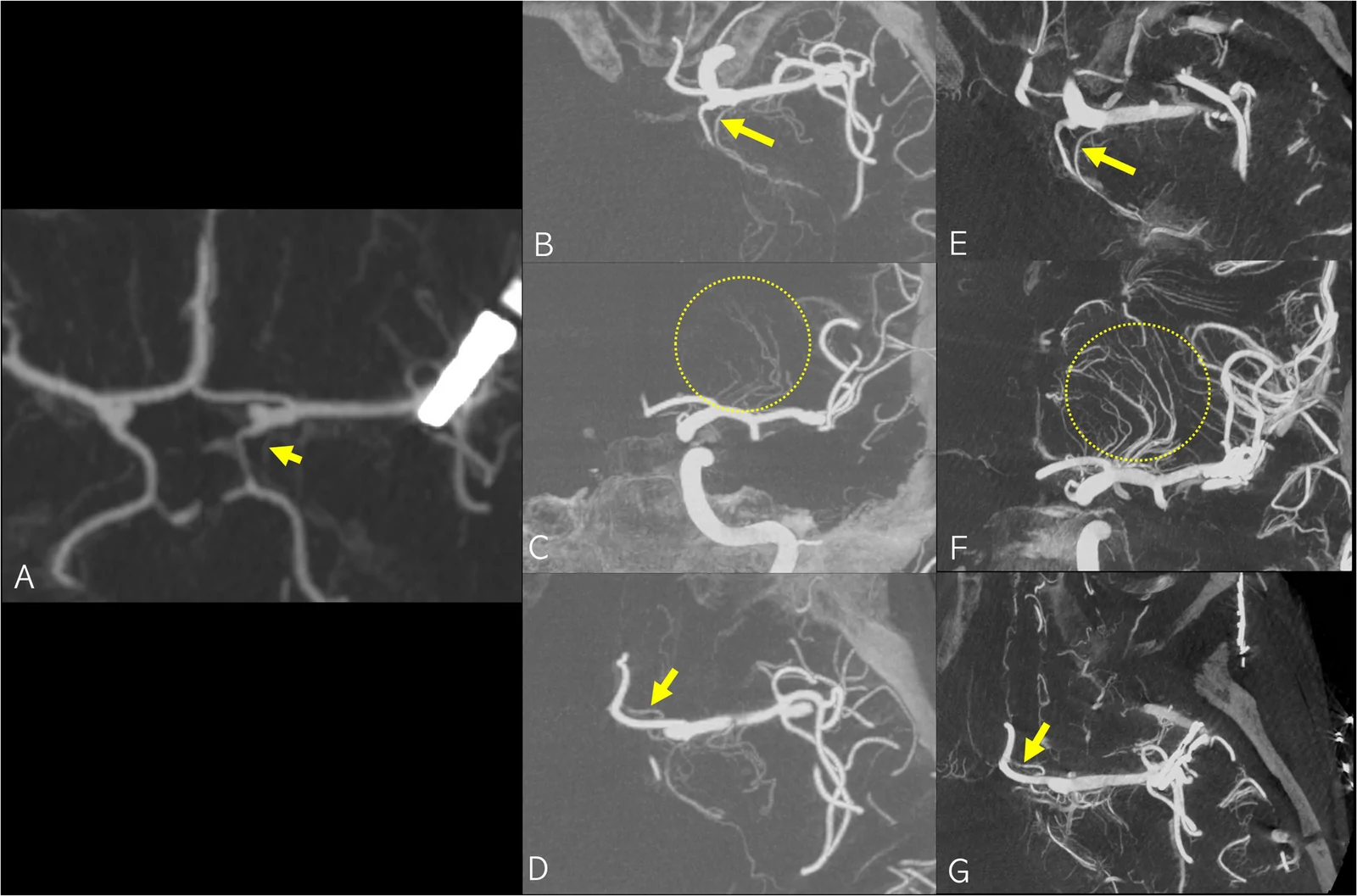
A woman in their 50s with a past medical history of traumatic brain injury and seizures presented for a diagnostic cerebral angiogram for multiple unruptured aneurysms found on computed tomography angiography (CTA). Axial preoperative CTA (**A**) at the level of the circle of Willis shows the left anterior choroidal artery (AChA, arrow) branching off the distal internal carotid artery. There is no visualization of the recurrent artery of Heubner (RAH) or the lateral lenticulostriate arteries (LSA). A prior left middle cerebral artery (MCA) bifurcation aneurysm clipping is noted. Left panel (B-D) depicts preoperative HR-CBCT. (**B**) Axial MIP shows the left AChA (arrow) branching off the distal internal carotid artery. (**C**) Coronal MIP shows the left LSA (dotted circle) branching off the left M1 segment. (**D**) Axial MIP shows the left RAH (arrow) branching off the left anterior cerebral artery (ACA). Right panel (**E**-**G**) depicts intraoperative HR-CBCT. (**E**) Axial MIP shows the left AChA (arrow). (**F**) Coronal MIP shows the left LSA (dotted circle) branching off the left M1 segment. (**G**) Axial MIP shows the left RAH (arrow) branching off the left ACA. HR-CBCT datasets were reconstructed with approximately 0.48 mm isotropic voxels (512 × 512 matrix), enabling high-resolution multiplanar visualization of small perforator arteries compared with CTA (0.625 mm slice thickness)

Patient demographics and operative characteristics were similar across imaging groups (Table S2). Differences in perforator visualization are demonstrated in example imaging from a single patient: preoperative CT angiography (Fig. 1A) shows limited perforator definition, whereas preoperative (Fig. 1B–D) and intraoperative HR-CBCT (Fig. 1E–G) demonstrate markedly improved visualization of small-caliber vessels.

**Fig. 2:**
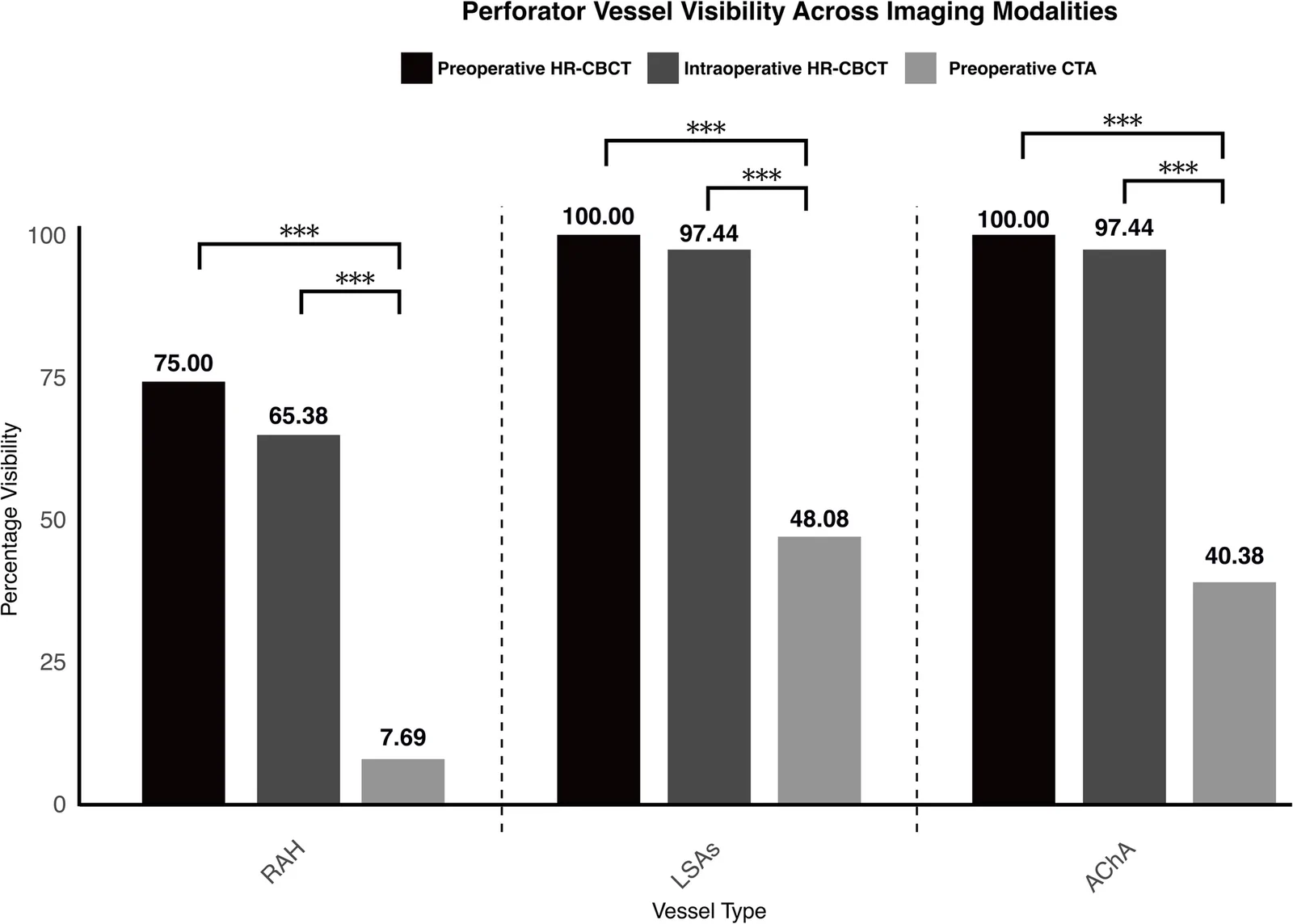
Neuroradiologists’ ability to visualize perforator vessels are represented as a bar plot. The x-axis represents each perforator vessel, the y–axis represents the percentage of total scans where that vessel can be identified, and the colors represent the different imaging modality. For each vessel, there is no significant difference between perforator vessel visualization in intraoperative and preoperative HR-CBCT, however there is a significant difference when comparing HR-CBCT to CTA. *** *p* < 0.001

Neuroradiologists were able to visualize perforator arteries more frequently on preoperative HR-CBCT than on preoperative CT angiography (Fig. 2). The anterior choroidal artery and lateral lenticulostriate arteries were identified in 100% of HR-CBCT scans, compared with 40.38% and 48.08%, respectively, on CT angiography. Visualization of the recurrent artery of Heubner was comparatively lower on HR-CBCT (75%) but remained substantially higher than on CT angiography (7.69%). When perforators were not visualized on preoperative imaging, the primary limiting factor was resolution rather than field-of-view (Table S3). On intraoperative HR-CBCT, by contrast, field-of-view limitations rather than resolution accounted for non-visualization of the LSAs in the small subset of cases where they were not seen, while resolution remained the dominant limit for the RAH (Table S3).

**Fig. 3:**
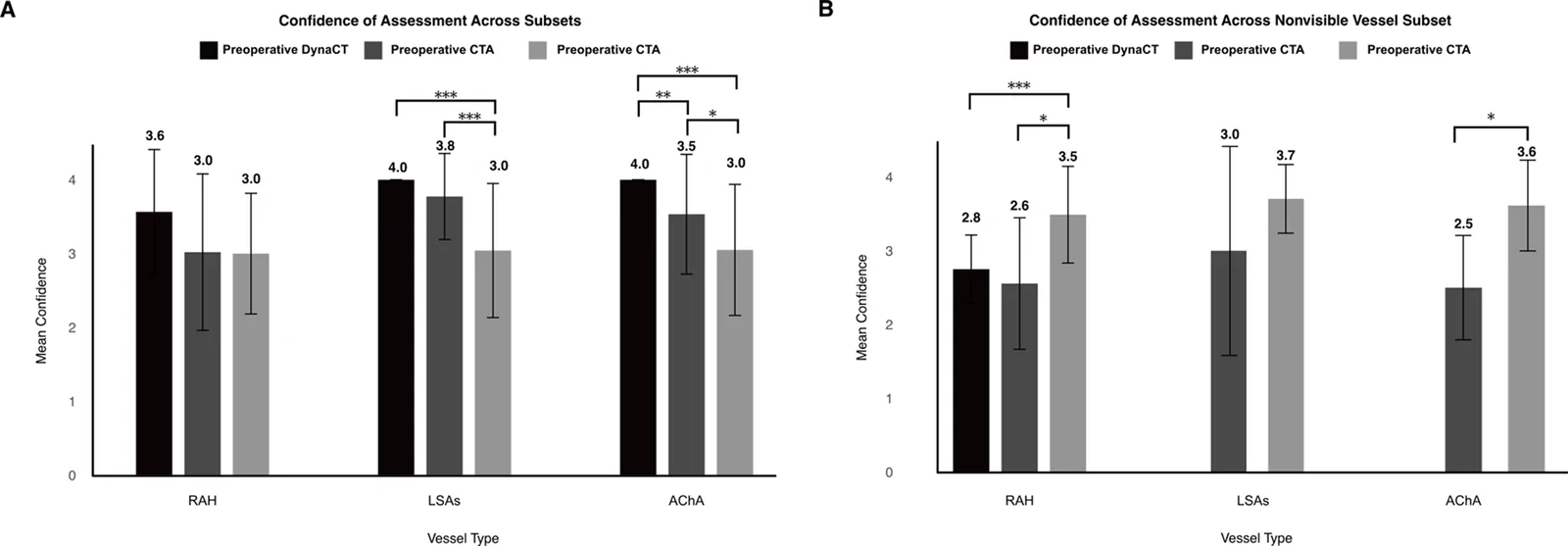
(**A**) Neuroradiologists’ confidence in correctly assessing perforator vessel visualization represented as a bar plot. The x-axis represents each perforator vessel, the y–axis represents the mean confidence score for each vessel, and the colors represent the different imaging modality. (**B**) Neuroradiologists’ confidence in correctly assessing non-visualization of perforator vessels due to resolution limitations. * *p* < 0.05, ** *p* < 0.01, *** *p* < 0.001

**Fig. 4:**
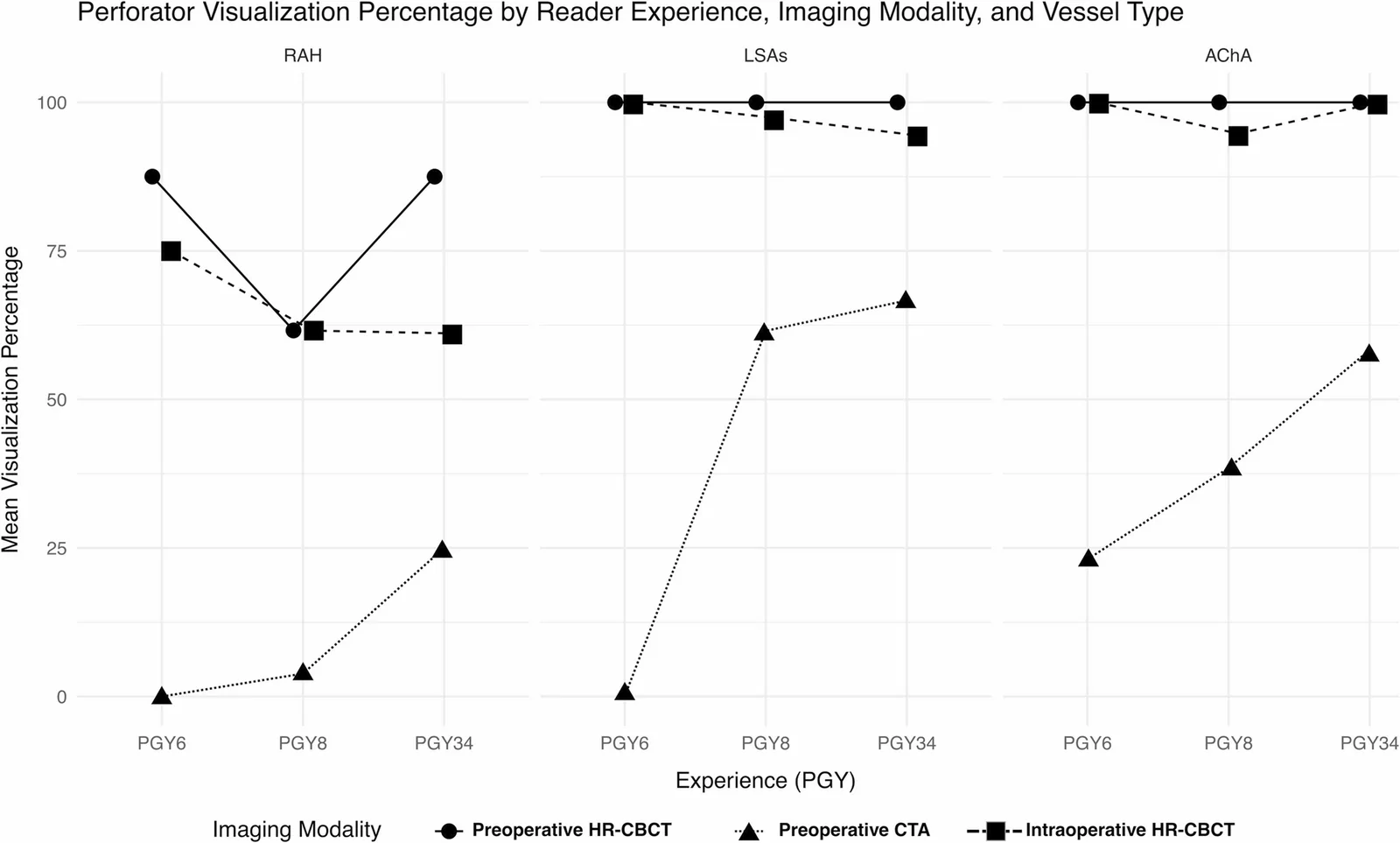
Neuroradiologists’ ability to visualize perforators as a function of their experience level and imaging modality is represented as a faceted line plot. The x-axis represents the experience of the reader (in terms of years after residency), the y–axis represents the mean visualization percentage. The different imaging modalities are separated by color and the different vessels are separated as subplots. Note that two neuroradiologists had the same number of years of experience (PGY-8) and the values shown are averages between the two

Not only were neuroradiologists able to better visualize perforators using HR-CBCT, but they also reported higher confidence when evaluating LSAs and AChA using HR-CBCT (Fig. 3A). When perforators were not visible, reader confidence was paradoxically higher on CTA than on HR-CBCT, reflecting readers’ recognition that CTA’s limited resolution was the expected cause of non-visualization. (Fig. 3B). Reader ability to visualize perforators largely remained consistent across experience levels for HR-CBCT, except for RAH, whereas CT angiography consistently demonstrated greater variability by neuroradiologist experience (Fig. 4).

**Fig. 5:**
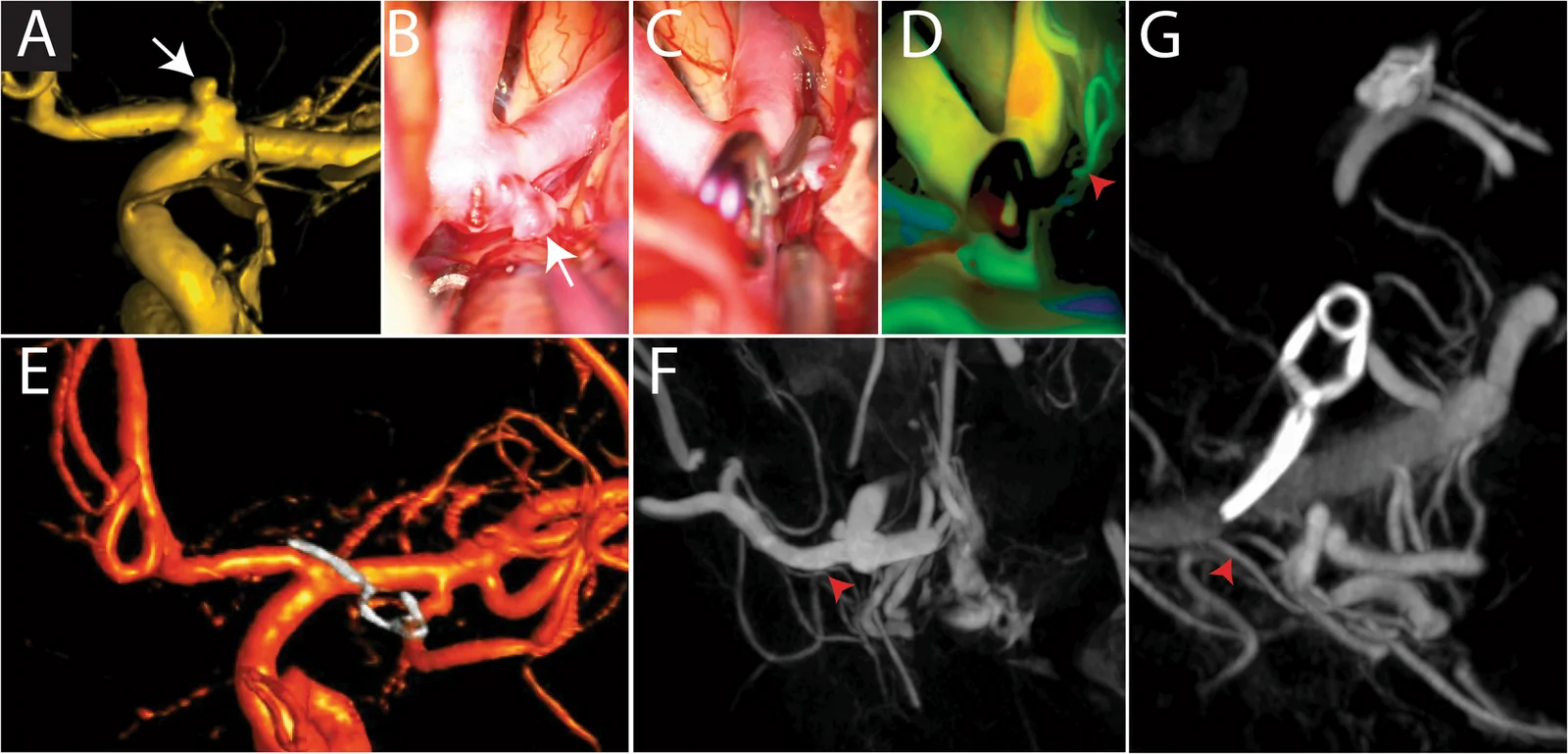
Use of High-Resolution Cone-Beam CT (DynaCT Micro) for perforator visualization during microsurgical clipping of a 5 mm dysplastic left ICA terminus aneurysm (white arrow) seen on preoperative 3D angiogram (**A**) and under microscope view (**B**). The aneurysm clip has obliterated the aneurysm dome (**C**) with perforator vessels (red arrowhead) seen in close proximity, appreciated on intraoperative indocyanine green angiography (**D**). An intraoperative rotational 3D angiogram shows no aneurysm filling with the aneurysm clip in place, but perforator anatomy cannot be appreciated (**E**). Preoperative (**F**) and intraoperative (**G**) High-Resolution Cone-Beam CT (DynaCT Micro) reconstructions provide excellent visualization of the corresponding perforator vessels (red arrowhead), confirming their patency

Comparison between preoperative and intraoperative HR-CBCT showed only a minimal reduction in perforator detection intraoperatively (Fig. 2). In the case of LSAs, the inability to visualize the vessel intraoperatively was caused by field-of-view limitations rather than resolution limitations (Table S3, Fig. 3B). The presence of intraoperative artifacts—including surgical clips, instruments, and missing bone flaps—did not meaningfully affect visualization of the RAH, AChA, or LSAs, nor did it reduce reader confidence (Figs. 3A and 5).

On intraoperative HR-CBCT, inter-reader agreement for vessel visibility was almost perfect for the AChA (AC1 = 0.947, *p* < 0.001) and LSAs (AC1 = 0.947, *p* < 0.001), while agreement for RAH visualization was moderate (AC1 = 0.593, *p* < 0.001), consistent with the greater difficulty of identifying this smaller perforating vessel due to its highly variable origin. On preoperative HR-CBCT, agreement was perfect for LSAs and AChA visibility while RAH visibility showed poor, non-significant agreement (AC1 = 0.266, *p* = 0.316; *N* = 8). On preoperative CTA, readers showed strong agreement for RAH visualization (AC1 = 0.851, *p* < 0.001), likely reflecting a consensus agreement of its difficult visualization. Agreement for the AChA (AC1 = 0.283) and LSAs (AC1 = − 0.101) was poor, indicating substantial disagreement about perforator visibility on conventional CTA.

For reader confidence, ICC [1, 2] values were uniformly low across all vessels and modalities (− 0.105 to 0.083), indicating poor absolute agreement in confidence scores. However, Gwet’s AC2 with quadratic weights, which accounts for the ordinal structure of the scale and penalizes larger disagreements more heavily, demonstrated substantial agreement for the LSAs (AC2 = 0.751, *p* < 0.001) and AChA (AC2 = 0.744, *p* < 0.001) on intraoperative HR-CBCT, as well as substantial agreement for the AChA (AC2 = 0.701, *p* < 0.001) and LSAs (AC2 = 0.677, *p* < 0.001) on preoperative CTA. Confidence agreement for the RAH was generally lower across modalities (AC2 range: 0.369–0.602), consistent with the challenge of resolving this vessel. On preoperative HR-CBCT, confidence agreement for the RAH reached moderate levels (AC2 = 0.602, *p* < 0.01). Lastly, there was no significant difference in the mean effective dose of radiation between CT angiography (1.92 ± 1.6 mSv) and HR-CBCT (1.48 ± 0.06 mSv; *p* = 0.700).

## Discussion

This study systematically compared three imaging modalities—preoperative HR-CBCT, intraoperative HR-CBCT, and conventional preoperative CT angiography (CTA)—to evaluate their ability to depict intracranial perforator arteries. Our findings demonstrate that both preoperative and intraoperative HR-CBCT substantially outperformed CTA in perforator visualization. More notably, we have also demonstrated that intraoperative scans achieved nearly equivalent performance to preoperative scans despite the potential for operative artifacts.

We found that preoperative HR-CBCT scans provided the most consistent benchmark for perforator visualization, particularly with the AChA and LSAs, which were visible in all cases, as well as for the basilar artery perforators (BAPs) and posterior cerebral artery perforators (PCAPs) in the relevant posterior circulation cases. The only exception was the recurrent artery of Heubner (RAH), which was not visualized in 25% of scans due to resolution and field-of-view limits. RAH is also a highly variable perforator vessel and can originate from the anterior cerebral artery, A1, A2, or the anterior communicating artery complex [4, 19, 20]. This variability could have contributed to neuroradiologists finding it consistently difficult to visualize RAH. Intraoperative HR-CBCT scans achieved nearly identical visibility rates, despite the presence of surgical artifacts such as surgical clips, retractors, skull clamp, presence/absence of bone flap, and a different imaging environment. Crucially, many intraoperative non-visualizations were attributable to field-of-view limitations rather than resolution (Table S3). This distinction underscores that the technical setup, rather than intrinsic image quality, accounted for missed vessels intraoperatively. By contrast, conventional CTA was inadequate for perforator assessment. In every instance of non-visualization, poor resolution was the limiting factor. Confidence ratings reflected this contrast: neuroradiologists were highly confident when perforators were not seen on CTA, recognizing the modality’s inherent resolution limits, but expressed lower confidence when vessels were absent on HR-CBCT (Table S3).

Our study is the first of its kind demonstrating that intraoperative HR-CBCT maintains perforator vessel visualization comparable to preoperative scans and drastically superior to preoperative CTA, further establishing its feasibility as a complementary intraoperative tool. These findings have important implications for cerebrovascular surgery. Intraoperative HR-CBCT can provide resolution comparable to preoperative scans, making it a practical tool for verifying perforator patency after aneurysm clipping. Prior work has shown the value of intraoperative HR-CBCT in detecting subtle device mal-apposition or under-expansion not seen on DSA, for example during flow-diverter implantation for unruptured intracranial aneurysms and stent implantation for chronic cerebral artery occlusion [21]. High-resolution CBCT has also been used to identify incomplete stent deployment and coil packing in aneurysm treatment, findings that directly altered management [22]. These experiences reinforce our finding that intraoperative CBCT can uncover issues invisible to conventional 2D-DSA and allow immediate corrective action. The ability to identify compromised flow intraoperatively raises the possibility of correcting interventions before irreversible ischemia occurs.

Beyond perforator assessment, intraoperative HR-CBCT also provided high-resolution depiction of parent vessel caliber after clip placement, allowing reassurance regarding parent vessel patency in cases where stenosis or compromise was a concern. This information complements intraoperative microscope-based techniques such as indocyanine green and fluorescein angiography, which depict only those perforators that lie within the immediate surgical field of view; perforators that course behind the parent vessel or are otherwise hidden from microscope view cannot be assessed with these techniques, whereas HR-CBCT enables their evaluation. Intraoperative 3D-RA can supplement understanding of residual aneurysm filling after microsurgical clipping compared to intraoperative 2D DSA alone [23]. Intraoperative HR-CBCT, which is essentially another rotational spin, can further supplement the information gained through intraoperative 2D DSA and intraoperative 3D-RA with an insight into perforator vessel patency [23]. 

Furthermore, preoperative HR-CBCT supplies a detailed anatomical roadmap of perforators, complementing DSA and allowing surgeons to anticipate the course of vessels in relation to surgical targets. This parallels evidence where HR-CBCT outperformed 3D-RA for perforator visualization around MCA aneurysms, influencing treatment decisions [10]. Similarly, HR-CBCT angiography has been integrated into AVM surgical planning and neuronavigation, underscoring the modality’s utility for preoperative strategy [24]. CTA, in contrast, fails to provide this level of detail and cannot reliably inform perforator-preserving strategies.

Prior studies have established the superiority of HR-CBCT over angiographic techniques in preoperative or diagnostic settings. HR-CBCT has been shown to offer significantly higher sensitivity and specificity for perforator visualization compared to 3D-RA in unruptured MCA aneurysms [10]. It has also provided much clearer perforator visualization than either 2D-DSA or 3D-RA in vertebral artery dissections, with imaging findings corresponding to infarct distributions [5]. Further, intraoperative contrast- enhanced CBCT has outperformed both 3D-RA and DSA in the setting of mechanical thrombectomy by allowing visualization of distal clot margins and downstream arteries, improving first-pass efficacy [25]. Reviews of HR-CBCT applications in the neuroangiography suite have highlighted its expanding role across aneurysms, AVMs, DAVFs, and stroke [12]. At the same time, new technical developments are pushing the limits of resolution even further. Ultra–high-resolution CBCT myelography has been shown to detect spinal CSF leaks invisible to digital subtraction myelography, suggesting a trajectory toward ever-smaller vessel and defect visualization [26]. Together, these studies highlight the unique strengths of HR-CBCT and suggest its potential to transform intraoperative vascular imaging. In addition, there is role for lenticulostriate perforator mapping for insular glioma surgery and there have been reports of MRA TOF utilization for this purpose [8]. Preoperative CBCT fused with insular glioma MRIs would provide the highest resolution map of LSA perforator for these cases with the ability to confirm their status post tumor resection from intraoperative HR-CBCT [8]. 

A potential criticism of the observed superiority of HR-CBCT in perforator vessel visualization compared to CTA is that the differences may be attributed to methodological factors to each modality, such as the intra-arterial versus intravenous route of contrast administration employed in HR-CBCT and CTA, or disparities in scanner technologies between institutions. However, recent studies have demonstrated that intravenous contrast delivery in CBCT can achieve similar levels of carotid artery filling density to intra-arterial contrast delivery in both canine and human models for cerebral perfusion [27]. Furthermore, we maintain that it is still appropriate to directly compare HR-CBCT and CTA as there have also been studies showing that CBCT with intravenous contrast maintains superiority in spatial resolution over CTA with intravenous contrast and that established protocols exist for optimizing intravenous delivery in CBCT [28, 29]. We would emphasize that the principal contribution of the present study is not a direct comparative analysis of HR-CBCT versus other imaging modalities, but rather the demonstration that intraoperative HR-CBCT achieves a similar degree of practical perforator vessel visualization compared to preoperative HR-CBCT. This study contains a few limitations: this study represents a single-center, retrospective feasibility analysis with a relatively small cohort, which limits generalizability and statistical power. The study was not designed to assess clinical endpoints, and no outcome data such as perforator infarction, ischemic complications, or functional neurological outcomes were analyzed. Accordingly, the findings should be interpreted as preliminary and focused on imaging performance rather than clinical efficacy. In addition, vessel visibility and reader confidence ratings were subjective and qualitative, rather than derived from objective measures of vessel delineation or sharpness such as spatial resolution, quantitative SNR, CNR, and vessel-to-background contrast metrics. Moreover, the study focused on selected perforators and generalization to other small vessels will require further validation. Practical barriers to broader adoption of HR-CBCT must also be acknowledged, including the need for a hybrid operating room or biplane angiography suite, the requirement for anesthesia coverage and intra-arterial catheterization for preoperative acquisitions, and operator and institutional expertise, all of which may limit feasibility outside high-volume cerebrovascular centers [12]. Furthermore, the present analysis did not include a direct head-to-head comparison between HR-CBCT and intraoperative two-dimensional DSA or three-dimensional rotational angiography, which are the current reference standards for intraoperative vessel assessment; such comparisons will be an important next step in establishing the relative value of HR-CBCT in operative workflows. The cohort did not include endovascularly coiled aneurysms; coil-related streak artifact may degrade HR-CBCT image quality, and the performance of HR-CBCT in coiled cases will require dedicated evaluation. In our cohort, one patient had pre-existing endovascular coils at the time of intraoperative HR-CBCT acquisition; in that case, coil-related streak artifact remained localized and did not preclude review of adjacent perforating arteries (Supplementary Figure S1). Finally, surgeon-perceived diagnostic confidence and prospective change-of-plan outcomes were not systematically captured in the present analysis and will be the focus of future prospective work.

Future studies should prospectively evaluate intraoperative HR-CBCT in multi-institutional settings, with standardized acquisition parameters to optimize field of view. Intraoperative DSA remains the gold standard for confirming aneurysm occlusion and large-vessel patency, but its limited resolution prevents reliable depiction of submillimeter perforators [1]. Head-to-head intraoperative comparisons between HR-CBCT and DSA are needed to determine to what extent HR-CBCT can match or surpass DSA for perforator assessment. Whether intravenous HR-CBCT can match the perforator-level visualization demonstrated here remains an open question that warrants prospective evaluation, particularly given the potential to reduce the need for intra-arterial catheterization. Moreover, longer-term applications, such as monitoring device endothelialization, may further expand the clinical role of HR-CBCT [21]. 

## Conclusions

Preoperative and intraoperative HR-CBCT provide superior visualization of intracranial perforator arteries compared with conventional CTA. Intraoperative HR-CBCT achieves performance comparable to preoperative scans, demonstrating its feasibility for real-time perforator assessment during cerebrovascular surgery. These findings support HR-CBCT as a valuable adjunct for intraoperative decision-making, with the potential to mitigate ischemic complications and improve surgical outcomes. While intraoperative DSA remains the current gold standard for vessel assessment, HR-CBCT offers complementary—and in the context of perforators, potentially superior spatial resolution. Future studies comparing intraoperative HR-CBCT to intraoperative DSA will be essential to determine its impact on surgical safety and outcomes.

## Supplementary Information

Below is the link to the electronic supplementary material.

Supplementary Material 1

## Data Availability

All data relevant to the study are included in the article or uploaded as supplementary information.
